# Detection of *PhoP*-mediated colistin resistance in Gram-negative bacteria without *mcr* genes in human population in the Ho Municipality, Ghana

**DOI:** 10.1016/j.heliyon.2024.e39633

**Published:** 2024-10-21

**Authors:** Emmanuel U. Osisiogu, Bhavana Singh, Patrick K. Feglo, Kwabena O. Duedu

**Affiliations:** a Department of Clinical Microbiology, College of Health Sciences, Kwame Nkrumah University of Science and Technology, Kumasi, Ghana; b Department of Science Laboratory Technology, Faculty of Applied Science and Technology, Dr Hilla Limann Technical University, Wa, Ghana; c Department of Paediatrics, University Health Services, Kwame Nkrumah University of Science and Technology, Kumasi, Ghana; d College of Life Sciences, Faculty of Health, Education and Life Sciences, Birmingham City University, Birmingham, United Kingdom; e Department of Biomedical Science, School of Basic and Biomedical Science, University of Health and Allied Sciences, Ho, Ghana

**Keywords:** Colistin resistance, *PhoP*, Gram-negative bacteria, Gut microbiota, Nanopore sequencing

## Abstract

**Introduction:**

Antimicrobial resistance (AMR) has become a global public health threat, with colistin emerging as a last-resort treatment option for multidrug-resistant Gram-negative infections. However, the emergence of colistin resistance, mediated by mechanisms like mutations in the *PhoP* gene, raises concerns about the future utility of this antibiotic. This study aimed to determine the prevalence of *PhoP*-mediated colistin resistance in Gram-negative bacteria isolated from the stool of residents in the Ho Municipality, Ghana.

**Methods:**

In this cross-sectional study, 110 stool samples were collected from June 2021 to December 2022. Gram-negative bacteria were isolated, and colistin susceptibility was determined by broth microdilution. Genomic DNA from resistant isolates was extracted and sequenced using the Nanopore platform to detect the presence of the *PhoP* gene.

**Results:**

Of the 107 Gram-negative isolates, 57 % were resistant to colistin. The *PhoP* gene was detected in 61.4 % of the colistin-resistant isolates, with the highest prevalence observed in *Proteus mirabilis*, *Escherichia coli*, *Pseudomonas aeruginosa*, and *Klebsiella pneumoniae*.

**Conclusion:**

The study reveals a high prevalence of *PhoP*-mediated colistin resistance among Gram-negative bacteria colonizing residents in the Ho Municipality, highlighting the role of the gut microbiota as a reservoir for antibiotic resistance. Continued surveillance and a collaborative One Health approach are crucial to address this growing threat.

## 1 Introduction

Antimicrobial resistance (AMR) has emerged as one of the greatest threats to global public health in recent years. The rapid spread of multidrug-resistant (MDR) Gram-negative bacteria, in particular, has led to a renewed interest in colistin as a last-resort treatment option. However, the emergence of colistin resistance, especially plasmid-mediated resistance via *mcr* genes, has raised concerns about the future utility of this antibiotic [1].

The increasing reports of colistin resistance, particularly mediated by the plasmid-borne *mcr* genes, threaten to compromise the efficacy of this antibiotic [2]. In addition to *mcr* genes, chromosomal mutations in regulatory genes such as *PhoP/PhoQ* and *PmrA/PmrB* can also confer colistin resistance [3]. The *PhoP/PhoQ* two-component system regulates genes involved in lipopolysaccharide (LPS) modification, which alters the bacterial cell surface charge and reduces colistin binding [4]. Mutations that constitutively activate the *PhoP/PhoQ* system can lead to colistin resistance [5]. The genomic basis of colistin resistance is complex and involves multiple pathways. The *mcr* genes encode phosphoethanolamine transferases that modify the lipid A component of lipopolysaccharide (LPS), reducing colistin binding to the bacterial outer membrane. On the other hand, the *PhoP/PhoQ* two-component system regulates genes involved in LPS modification, which alters the bacterial cell surface charge and reduces colistin binding [4]. Mutations that constitutively activate the *PhoP/PhoQ* system can lead to colistin resistance [5].

While much attention has focused on *mcr* genes, the role of *PhoP* in mediating colistin resistance in clinical and community settings is less well studied, particularly in Africa. Some studies have reported *PhoP* mutations in colistin-resistant *Klebsiella pneumoniae* isolates from hospitalized patients in Africa [5], but data on its prevalence in the community gut microbiota is lacking.

Although the emergence and dissemination of *mcr*-mediated colistin resistance have received significant attention, the prevalence and impact of *PhoP*-mediated resistance, particularly in community settings, are relatively understudied. Previous research has identified *PhoP* mutations as a mechanism of colistin resistance in clinical isolates of *K. pneumoniae* from hospitalized patients in some African countries [5]. However, data on the prevalence of *PhoP*-mediated resistance in the community gut microbiota, which can serve as a reservoir for antibiotic resistance genes, is limited.

The *PhoP/PhoQ* two-component regulatory system plays a crucial role in bacterial virulence and antimicrobial peptide resistance, including resistance to colistin. *PhoP* is the response regulator that, when phosphorylated by its sensor kinase *PhoQ*, activates or represses various genes involved in lipopolysaccharide (LPS) modifications [6]. These modifications can lead to reduced binding of cationic antimicrobial peptides like colistin to the bacterial outer membrane [7].

Research on *PhoP*-mediated colistin resistance has been conducted in various bacterial species. In *K. pneumoniae*, mutations in the *mgrB* gene, a negative regulator of the *PhoP/PhoQ* system, have been associated with colistin resistance [8]. Similarly, in *Salmonella enterica*, activation of the *PhoP/PhoQ* system has been linked to increased survival in the presence of antimicrobial peptides [9]. In *Pseudomonas aeruginosa*, a study has demonstrated that the *PhoP/PhoQ* system regulates the expression of genes involved in LPS modification, directly impacting susceptibility to polymyxins like colistin [10]. Furthermore, research has shown that mutations in *PhoQ* can promote lipid A modifications and polymyxin resistance in *P. aeruginosa* isolates from cystic fibrosis patients treated with colistin [11].

The human gut microbiota plays a crucial role in the dissemination of antibiotic resistance genes, as it serves as a reservoir for these genes, which can be transferred to pathogenic bacteria [12]. Despite these findings, there is a paucity of data on the prevalence and impact of *PhoP*-mediated colistin resistance in community settings, particularly in Africa. Most studies have focused on clinical isolates, leaving a significant gap in our understanding of the distribution and significance of this resistance mechanism in the general population.

This study specifically focused on colistin resistance due to its critical importance as a last-resort antibiotic. Understanding the dynamics of colistin resistance is particularly vital given its role as one of the few remaining effective treatments for infections caused by multidrug-resistant Gram-negative bacteria. Therefore, understanding the prevalence and distribution of resistance mechanisms, such as *PhoP*-mediated colistin resistance, in the community gut microbiota is essential for devising effective strategies to combat antimicrobial resistance. This study aimed to determine the prevalence of colistin resistance in Gram-negative bacteria isolated from the stool of residents in the Ho Municipality, Ghana. The findings of this study provide valuable insights into the role of the gut microbiota in harbouring and potentially disseminating colistin resistance.

## 2 Methodology

### 2.1 Study Design, study area and sample distribution

**Fig. 1:**
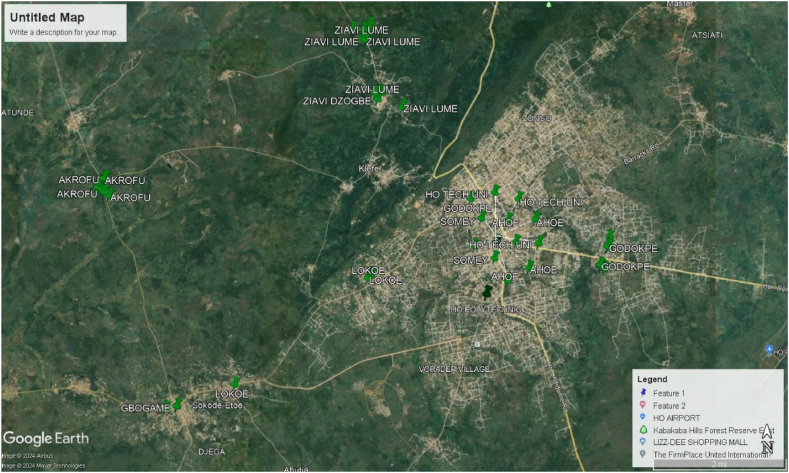
Geospatial distribution of water samples collected.

This cross-sectional study was conducted in Ho, the capital town of the Volta Region of Ghana. Ho was selected as the study site due to its representative urban population and access to diverse communities. A total of 110 stool samples were collected from consenting participants across various localities in Ho (Fig. 1) from June 2021 to December 2022. Ethical approval was obtained from the Committee on Human Research, Publications and Ethics of Kwame Nkrumah University of Science and Technology (CHRPE/AP/371/20).

### 2.2 Sample collection, isolation and identification of isolates

Stool samples were collected from both children and adults in selected households using sterile, leak-proof containers following standard protocols [13] and transported to the laboratory in cold boxes maintained at 4–8 °C [14] within 2 h of collection. Upon receipt, samples were streaked onto MacConkey agar and incubated aerobically at 37 °C for 18–24 h. To suppress the growth of normal flora and facilitate the isolation of potential pathogens, samples were initially cultured on MacConkey agar supplemented with vancomycin (10 μg/mL) and amphotericin B (2 μg/mL). This combination of antibiotics inhibited the growth of most Gram-positive bacteria and yeasts respectively. Lactose fermenting and non-lactose fermenting colonies were picked and sub-cultured on nutrient agar. Pure isolates were identified by Gram staining and standard biochemical tests [15].

### 2.3 Phenotypic colistin susceptibility testing

The colistin susceptibility of isolates was assessed using the broth microdilution method, following the guidelines set by the Clinical and Laboratory Standards Institute [16]. A colistin sulphate (Sigma-Aldrich) stock solution was prepared at 5120 μg/mL in sterile distilled water, filter-sterilized, and stored in aliquots at −20 °C. This stock was diluted to create a 256 μg/mL working solution in cation-adjusted Mueller-Hinton broth (CA-MHB). A series of two-fold dilutions were then made to achieve final concentrations ranging from 0.125 to 64 μg/mL.

Bacterial inocula were prepared by adjusting isolates to a 0.5 McFarland standard in sterile saline, followed by a 1:150 dilution in CA-MHB to reach approximately 5 × 10^5^ CFU/mL. The assay was performed in 96-well microtiter plates, with 100 μL of each colistin dilution added to wells in columns 1–10. Growth and sterility controls were included in columns 11 and 12, respectively.

After incubation at 35 ± 2 °C for 18–24 h, wells were examined for visible growth. The minimum inhibitory concentration (MIC) was determined as the lowest colistin concentration that completely inhibited visible bacterial growth. Susceptibility was interpreted based on CLSI breakpoints, with MIC ≤2 μg/mL considered susceptible and MIC >2 μg/mL resistant.

Quality control was ensured by including *Escherichia coli* ATCC 25922 and *P. aeruginosa* ATCC 27853 in each testing batch, with acceptable MIC ranges of 0.25–2 μg/mL and 0.5–4 μg/mL, respectively. Results were reported as MIC values along with their interpretation according to CLSI guidelines [16].

### 2.4 Detection of colistin resistance gene by polymerase chain reaction

Polymerase chain reaction (PCR) was utilized to identify the presence of *mcr* genes (*mcr*-1 through *mcr*-10) in colistin-resistant bacterial isolates obtained from stool samples. The PCR reaction mixture was prepared using a commercially available master mix containing Taq polymerase, buffer, and dNTPs. Primers specific to each *mcr* gene were reconstituted in nuclease-free water to create stock solutions, which were then diluted to working concentrations. The PCR reaction was set up according to the manufacturer's protocol, with extracted bacterial DNA serving as the template. To enhance efficiency, a multiplex PCR approach was adopted, allowing for the simultaneous detection of multiple *mcr* genes in a single reaction. Hence, two master mixes containing five primer combinations were prepared for each DNA extracted.

Thermocycling was performed using an Eppendorf thermocycler (Germany) with the following conditions for *mcr-1* to *mcr-5*: initial denaturation at 94 °C for 15 min; 30 cycles of denaturation at 94 °C for 30 s, annealing at 58 °C for 90 s, and extension at 72 °C for 1 min/kb; final extension at 72 °C for 10 min; and a final hold at 4 °C for 10 min, while *mcr-6* to *mcr-10* had the following conditions: initial denaturation at 95 °C for 3 min; 30 cycles of denaturation at 95 °C for 30 s, annealing at 55 °C for 90 s, and extension at 72 °C for 1 min/kb; final extension at 72 °C for 10 min; and a final hold at 4 °C for 10 min. The annealing temperature for some primers were slightly adjusted (52 °C for *mcr-6*, 50 °C for *mcr-7* and 53 °C for *mcr-8*) based on manufacturers instruction during the setup process. This was achieved using gradient PCR.

Primer pairs specific for *mcr*-1 to *mcr*-10 genes were used, with their sequences and expected product sizes detailed in Table 1. Following amplification, PCR products were separated by agarose gel electrophoresis to visualize and identify specific *mcr* genes based on their characteristic band sizes. Each PCR run included appropriate positive and negative controls to ensure result validity and reliability. Positive controls for *mcr* genes were obtained using in-house generated strains of *E. coli* [17].

**Table 1:** Primers for multiplex-PCR.

Primer name	Sequence (5′-3′)	Target gene	Size (bp)	Reference
*mcr-1*_320bp_fw	AGTCCGTTTGTTCTTGTGGC	*mcr*-1	320	[18]
*mcr-1*_320bp_rev	AGATCCTTGGTCTCGGCTTG	*mcr*-1	320	[18]
*mcr-2*_700bp_fw	CAAGTGTGTTGGTCGCAGTT	*mcr*-2	715	[18]
*mcr-2*_700bp_rev	TCTAGCCCGACAAGCATACC	*mcr*-2	715	[18]
*mcr-3*_900bp_fw	AAATAAAAATTGTTCCGCTTATG	*mcr*-3	929	[18]
*mcr-3*_900bp_rev	AATGGAGATCCCCGTTTTT	*mcr*-3	929	[18]
*mcr- 4*_1100bp_fw	TCACTTTCATCACTGCGTTG	*mcr*-4	1,116	[18]
*mcr-4*_1100bp_rev	TTGGTCCATGACTACCAATG	*mcr*-4	1,116	[18]
*mcr*-*5*_fw	ATGCGGTTGTCTGCATTTATC	*mcr*-5	1,644	[18]
*mcr-5*_rev	TCATTGTGGTTGTCCTTTTCTG	*mcr*-5	1,644	[18]
*mcr-*6_mp_fw	AGCTATGTCAATCCCGTGAT	*mcr*-6	252	[19]
*mcr*-6_mp_rev	ATTGGCTAGGTTGTCAATC	*mcr*-6	252	[19]
*mcr*-7_mp_fw	GCCCTTCTTTTCGTTGTT	*mcr-*7	551	[19]
*mcr*-7_mp_ rev	GGTTGGTCTCTTTCTCGT	*mcr*-7	551	[19]
*mcr*-8_mp_fw	TCAACAATTCTACAAAGCGTG	*mcr*-8	856	[19]
*mcr*-8_mp_ rev	AATGCTGCGCGAATGAAG	*mcr*-8	856	[19]
*mcr*-9_mp_fw	TTCCCTTTGTTCTGGTTG	*mcr*-9	1011	[19]
*mcr*-9_mp_ rev	GCAGGTAATAAGTCGGTC	*mcr*-9	1011	[19]
*mcr*-10_fw (090065_up_SacI)	AAAAAAGAGCTCTCCGCTTTGTATCCCAATAC	*mcr-*10	1620	[20]
*mcr-10*_rev (090065_down_EcoRI)	AAAAAAGAATTCTTTTATAATTT CCGGCAGCA	*mcr-*10	1620	[20]

### 2.5 Detection of colistin resistance gene by whole genome nanopore sequencing

Genomic DNA was isolated from colistin resistant bacteria cultured from stool samples using the LBP Nucleic acid extraction and purification kit (Guangzhou LBP Medical Modified Science and Technology Co.Ltd). DNA quantity was measured using a fluorometric method with a Qubit 4 Fluorometer (Thermo Fisher Scientific, USA), while quality was assessed using a Nanodrop One Spectrophotometer (Thermo Fisher Scientific, USA), following the manufacturers' guidelines.

Library preparation for sequencing was performed using the Rapid Barcoding Kit 96 (SQK-RBK110.96), and sequencing was conducted on a MinION Mk1C device with a Spot-ON Flow Cell (FLO-MIN106D R9.4.1 Version), all from Oxford Nanopore Technologies.

Raw sequencing data in fast5 format was converted to fastq format using the guppy basecaller package with the following command:

guppy_basecaller -i fast5 -s fastq --flowcell FLO-MIN106 --kit SQK-RBK110-96 -x cuda:all

Where -i specifies the directory containing the input fast5 files.

Demultiplexing of fastq reads was accomplished using the guppy barcoder package with this command:

guppy_barcoder -i pass -s barcodes --barcode_kits SQK-RBK110-96 - x cuda:all

Where -i indicates the "pass" directory containing the basecalled reads.

Initial identification of colistin resistance genes was performed using the EPI2ME cloud-based platform via the EPI2ME desktop agent software. Two workflows were employed: FASTQ WIMP for species identification and quantification, and FASTQ Antimicrobial Resistance for AMR profiling. The "detect barcode" option was disabled, and results were sorted into folders corresponding to the original barcode names.

The Resistance Gene Identifier (RGI) tool and the Comprehensive Antibiotic Resistance Database (CARD) [21] were used for further analysis and confirmation of colistin resistance genes in the samples.

Read polishing was performed using Porechop following read concatenation:

cat barcode∗/∗.fastq > barcode∗_merged.fastq

porechop -i /barcode∗_merged.fastq -o barcode∗_trimmed.fastq

Where -i specifies the directory containing the merged barcoded reads.

For selected barcodes, consensus sequences were constructed using Canu with the following command:

canu -d /path/to/output_directory -p output_file_name genomeSize = "4.8m" maxInputCoverage = 10000 corOutCoverage = 10000 corMhapSensitivity = high corMinCoverage = 0 redMemory = 32 oeaMemory = 32 batMemory = 60 -nanopore /path/to/merged_input_reads

Parameters were set to default values or adjusted based on the available computing resources.

The resulting contigs were analysed using BlastN for alignment and potential ARG identification against the NCBI nucleotide collection database. Detailed scripts used in the post-sequencing analysis are provided in the supplementary material.

### 2.6 Data analysis

Data were analysed using SPSS Statistics v26 (IBM). Descriptive statistics were used to summarize the prevalence of colistin resistance and the presence of the *PhoP* gene among isolates. The phenotypic prevalence of colistin resistance was calculated as the percentage of isolates with MIC >2 μg/mL out of the total number of isolates tested. For genetic analysis, the prevalence of the *PhoP* gene was determined separately for phenotypically resistant and susceptible isolates. Chi-square tests were used to compare the prevalence of the *PhoP* gene between these two groups, as well as across different bacterial species, gender, and age groups.

The distribution of *PhoP-*positive isolates across different bacterial species was presented as percentages. Age-related patterns in *PhoP* carriage were examined by calculating the percentage of *PhoP*-positive isolates within defined age categories.

To assess the relationship between phenotypic resistance and genotypic markers, the prevalence of the *PhoP* gene was compared between isolates classified as resistant or susceptible based on MIC testing. For all statistical tests, a P-value <0.05 was considered statistically significant. Graphs and charts were created to visually represent the distribution of resistant isolates and *PhoP* gene carriage across different variables.

## 3 Results

**Fig. 2:**
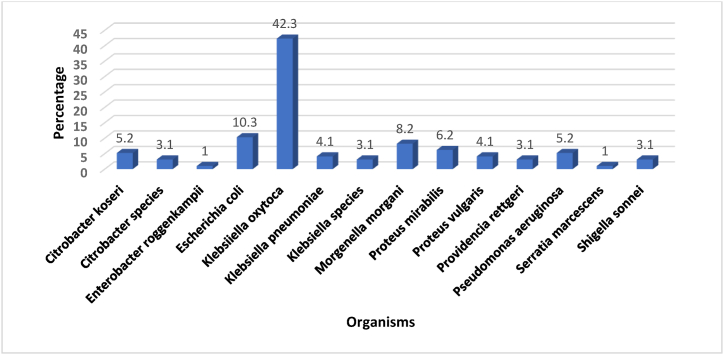
Organisms isolated and identified using culture and biochemical testing method.

Out of the 110 stool samples analysed, 107 yielded Gram-negative bacterial growth of which 97 were identified via standard biochemical testing methods. The most common bacteria isolated were *Klebsiella species*, *E. coli*, *Morgenella morgani,* and *Proteus mirabilis* (Fig. 2).

**Fig. 3:**
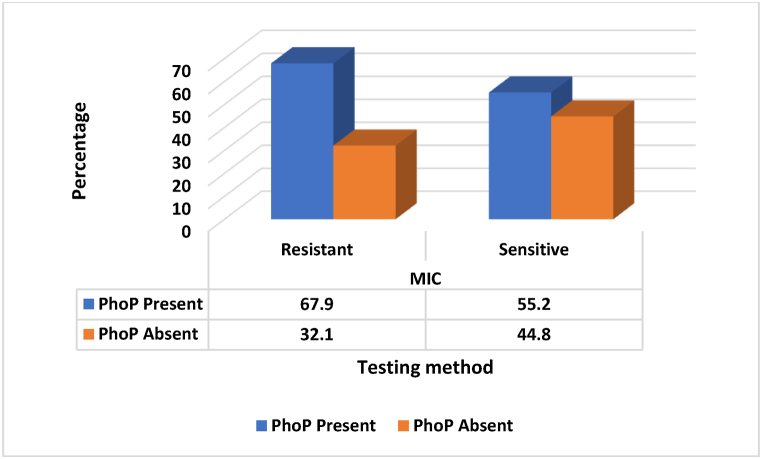
Comparison between phenotypic and genotypic susceptibility testing methods.

MIC testing was conducted on 107 stool samples to detect colistin resistance. Results showed that 61 out of 107 isolates (57 %) exhibited phenotypic resistance to colistin. No *mcr* genes were detected in any of the isolates from this study. For further genetic analysis, a subset of isolates was selected: 28 MIC-resistant and 29 MIC-susceptible isolates. These underwent whole genome sequencing to detect the presence of colistin resistance genes. Sequencing results revealed that among the MIC-resistant isolates, 19 out of 28 (67.9 %) carried the *PhoP* gene. However, among the MIC-susceptible isolates, 16 out of 29 (55.2 %) carried the *PhoP* gene (Fig. 3).

**Fig. 4:**
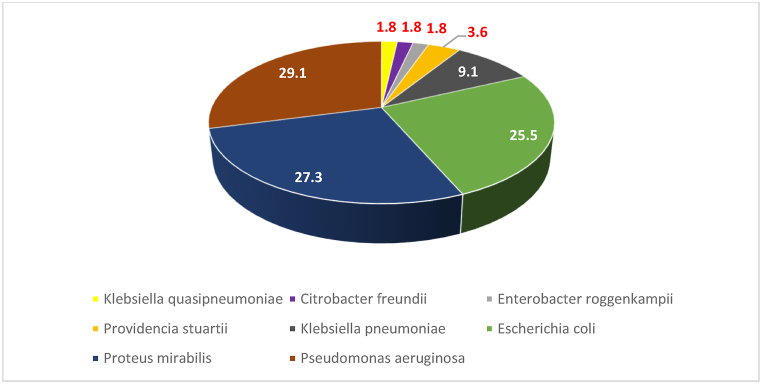
Percentage frequency of Isolates with *PhoP* gene.

The *PhoP* gene was most frequently found in *P. mirabilis*, *E. coli*, *P. aeruginosa*, and *K. pneumoniae* (Fig. 4).

**Fig. 5:**
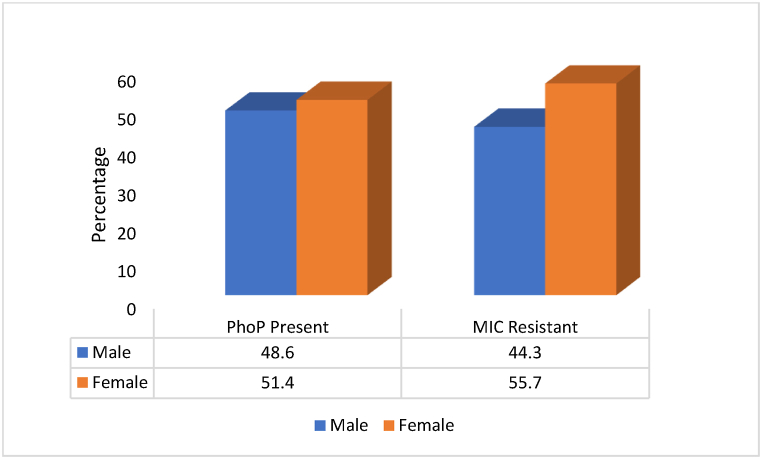
Gender variation in Resistance profile per testing method.

Although colistin resistance was more prevalent in females, there was no significant difference (p > 0.05) in the prevalence of *PhoP* in organisms isolated from males (48.6 %) compared to females (51.4 %) (Fig. 5).

**Fig. 6:**
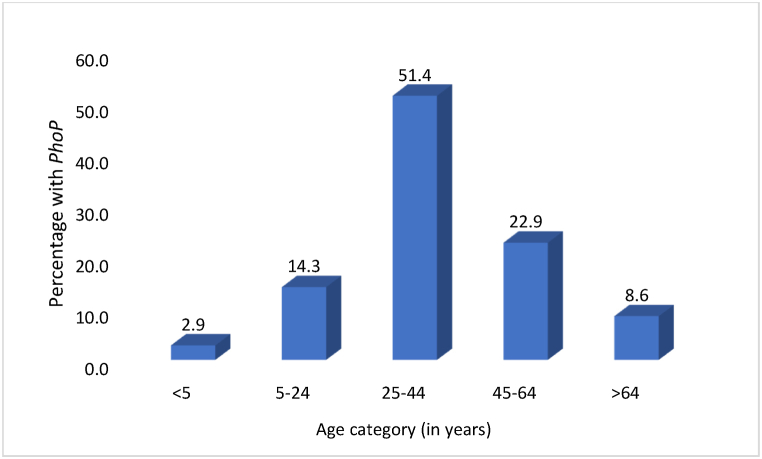
Distribution of organisms possessing *PhoP* across various age categories.

Age-wise distribution showed *PhoP* carriage was highest in the 25–44-year group, with 51.4 % representing isolates from this demographic harbouring the gene (Fig. 6).

## 4 Discussion

This study found a high prevalence (57 %) of colistin resistance among Gram-negative bacteria isolated from stool samples in the Ho Municipality of Ghana, contrasting sharply with previous reports of low resistance rates (0.67 %) among enterobacteria [22]. This significant difference between the two prevalence could be attributed to several factors, including geographical variations in antibiotic use practices, differences in study populations (clinical as against community), changes in resistance patterns over time, methodological differences, and the broader range of Gram-negative bacteria included in this current study. The prevalence of colistin resistance reported in this current study is however consistent with the growing concern about the emergence and spread of colistin resistance in various settings, including the community [23]. It is important to note that our study was conducted in the Ho Municipality, which is home to several healthcare facilities, including the Ho Teaching Hospital. The proximity of our sampling locations to these medical centres, and the potential connection between community sewage systems and hospital waste, may have influenced our findings. Hospital effluents are known reservoirs of antibiotic-resistant bacteria and resistance genes [24]. The potential mixing of hospital and community wastewater could facilitate the spread of resistant organisms and mobile genetic elements into the wider community [25]. This urban setting with its healthcare infrastructure may partly explain the high prevalence of colistin resistance observed in our study. Contrary to several studies reporting *mcr* genes in colistin-resistant isolates sources [26,27], our study detected no *mcr* genes, prompting further investigation via next-generation sequencing to identify alternative resistance mechanisms. The absence of *mcr* genes in our samples suggests that colistin resistance in this population may be primarily mediated by other mechanisms, such as chromosomal mutations like those in the *PhoP* gene. This highlights the importance of considering multiple resistance mechanisms when investigating colistin resistance and emphasizes the need for comprehensive genetic analysis beyond targeted PCR approaches. The lack of *mcr* genes also raises questions about the local patterns of antibiotic use and resistance dissemination, which may differ from other regions where *mcr* genes are more prevalent.

The detection of the *PhoP* gene in 67.9 % of the colistin-resistant isolates (Fig. 3) highlights the significant role of this chromosomal resistance mechanism in mediating resistance to this last-resort antibiotic. Our observed 67.9 % prevalence of *PhoP*-mediated colistin resistance significantly exceeds the 0.5–35 % range reported in a systematic review of global *mcr*-mediated resistance rates from 1980 to 2018 [28]. However, a review on the global spread of *mcr* genes noted that while prevalence varies widely, some studies have reported alarmingly high rates in certain jurisdictions, such as 64.9 % *mcr-1* positivity in *E. coli* from chicken in China [29]. Another study in China found high rates of *mcr-1* positive *Enterobacteriaceae* in human faecal samples, with carriage rates of up to 36 % in some regions [26]. While not directly comparable to the 61.4 % resistance rate, it demonstrates the potential for high prevalence of colistin resistance genes in community settings. These two studies suggests that very high rates of colistin resistance, similar to the 61.4 % found in this current study, are possible in some contexts. Although the *PhoP* gene was more prevalent in phenotypically resistant isolates (Fig. 3), its presence alone doesn't guarantee colistin resistance, suggesting a complex interplay of genetic and possibly environmental factors in resistance development.

The presence of the *PhoP* gene was most frequently observed in *P. mirabilis*, *E. coli*, *P. aeruginosa*, and *K. pneumoniae* (Fig. 4). These findings are in line with previous studies that have reported *PhoP*-mediated colistin resistance in these species [5]. For instance, a global emergence of colistin resistance in *K. pneumoniae* from healthy humans and patients has been reported in various countries, including Thailand, Israel, Nigeria, and France, owing to inactivation of the *PhoP/PhoQ* regulator *mgrB* [5]. The high prevalence of *PhoP* in key pathogens like *E. coli* and *K. pneumoniae* is particularly alarming, as these organisms are a major cause of both community-acquired and healthcare-associated infections [30].

More so, the presence of *PhoP*-mediated colistin resistance in *P. aeruginosa* is alarming, as this opportunistic pathogen is notorious for its intrinsic resistance to multiple antibiotics and its ability to acquire additional resistance mechanisms [31,32]. The detection of *PhoP* in *P. aeruginosa* in this study underscores the potential for this species to develop resistance to even last-resort antibiotics like colistin. This is supported by a study which reported the rapid and consistent evolution of colistin resistance in extensively drug-resistant *P. aeruginosa* during morbidostat culture [33]. Another study demonstrated that mutations in the *PhoPQ* regulatory system, particularly loss-of-function mutations in *PhoQ*, can confer high-level resistance to polymyxins (including colistin) in clinical isolates of *P. aeruginosa* from cystic fibrosis patients (Miller et al., 2011). Their study showed that these mutations promote lipid A modifications that reduce colistin binding, can occur independently of other known resistance mechanisms, and result in extremely high levels of resistance (MICs >512 mg/L in some cases), thus highlighting *P. aeruginosa's* potential to develop resistance to this last-resort antibiotic through *PhoP*-mediated mechanisms.

There was no significant difference in the prevalence of *PhoP* between males and females, although resistance was higher in females (Fig. 5). This finding suggests that factors other than gender may influence the acquisition and expression of colistin resistance. A study also found colistin to be less effective in males than in females [34]. In contrast, a study by Zhong et al. (2018) found higher rates of *mcr*-1-positive multidrug-resistant *Enterobacteriaceae* in faecal carriage among females compared to males in China. The discrepancy in gender-related findings between the present study and that of Zhong et al. (2018) as well as Hossain et al. (2020) may be attributed to differences in the specific resistance mechanism investigated (*PhoP* vs. *mcr*-1) and the geographical setting.

*PhoP* prevalence peaked in the 25–44 year age group (Fig. 6), potentially impacting community spread and emphasizing the need for targeted interventions in this economically productive population in this region. Similarly, the highest rates of *mcr*-1 carriage among adults aged 19–45 years was documented in China [26], emphasizing the importance of monitoring and controlling the spread of colistin resistance in this age group. It's worth noting that the consistency between our findings and those of Zhong et al. (2018) suggests that this age-related pattern of colistin resistance gene carriage might be a broader trend, potentially related to factors such as antibiotic use patterns, occupational exposures, or social behaviours in this age group.

The prevalence of *PhoP* in gut microbiota species indicates potential for wider dissemination of colistin resistance [12], supported by studies detecting resistance genes in diverse environmental settings including water sources [35] and imported vegetables [36].The high prevalence of *PhoP* in diverse species in this study emphasizes the importance of community-based surveillance to monitor the emergence and spread of resistance determinants [5].

The role of the gut microbiota in harbouring and potentially disseminating antibiotic resistance genes is further highlighted by studies investigating the prevalence of *mcr* genes in human faecal samples. For example, high rates of human faecal carriage of *mcr*-1-positive multidrug-resistant *Enterobacteriaceae* have been reported in China, with a prevalence of 15.2 % among healthy individuals [26]. Similarly, a systematic review and meta-analysis found a pooled worldwide prevalence of 3.0 % for *mcr*-mediated colistin-resistant *E. coli* in isolates from clinical samples and healthy humans [27]. These findings underscore the importance of the human gut as a reservoir for colistin resistance genes and the potential for their dissemination in the community.

Several studies have reported the occurrence of colistin resistance in animals, including pigs [37] and poultry [38], underlining the potential for transmission between animals and humans. For instance, there have been reports of clonal transmission of a colistin-resistant *E. coli* from a domesticated pig to a human in Laos, highlighting the potential for zoonotic transmission of resistant strains [37]. The study's findings also highlight the need for a One Health approach to tackle the growing threat of antibiotic resistance [39]. This approach recognizes the interconnectedness of human, animal, and environmental health and emphasizes the need for collaborative efforts to address the problem.

## 5 Conclusion

This study reveals a high prevalence of *PhoP*-mediated colistin resistance among Gram-negative bacteria in the Ho Municipality of Ghana, clearly without detecting *mcr* genes. The presence of *PhoP* in diverse species, including key pathogens like *E. coli* and *K. pneumoniae*, highlights the gut microbiota's role as a reservoir for antibiotic resistance. These findings suggest that colistin resistance in this community is primarily mediated by chromosomal mechanisms rather than plasmid-borne genes, emphasizing the need for comprehensive genetic analysis in resistance investigations. The results highlight the urgency for continued surveillance, molecular characterization of resistance mechanisms, and a One Health approach integrating human, animal, and environmental health. Further research is needed to understand the clonal relatedness of *PhoP*-positive isolates, their potential for horizontal gene transfer, and factors contributing to high colistin resistance prevalence in this setting.

## CRediT authorship contribution statement

**Emmanuel U. Osisiogu:** Writing – original draft, Investigation, Conceptualization. **Bhavana Singh:** Writing – review & editing, Supervision, Conceptualization. **Patrick K. Feglo:** Writing – review & editing, Supervision, Conceptualization. **Kwabena O. Duedu:** Writing – review & editing, Supervision, Conceptualization.

## Ethics approval

This study forms part of a larger study that received ethical clearance from the Committee on Human Research, Publications and Ethics of Kwame Nkrumah University of Science and Technology (KNUST)- Ghana, with reference number (CHRPE/AP/371/20).

## Data and code availability statement

Data included in article/supplementary material is referenced in the article.

## Funding

This work was supported by the research facilities provided by Tractilis BioLabs and Duedu Laboratory. The authors have no additional funding sources to disclose.

## Declaration of competing interest

The authors declare the following financial interests/personal relationships which may be considered as potential competing interests:Osisiogu U. Emmanuel reports equipment, drugs, or supplies was provided by Tractilis biolabs, and Duedu laboratory. If there are other authors, they declare that they have no known competing financial interests or personal relationships that could have appeared to influence the work reported in this paper.
