# Supplementary material for: Detection of PhoP-mediated colistin resistance in Gram-negative bacteria without mcr genes in human population in the Ho Municipality, Ghana
Source: Heliyon. 2024 Oct 21;10(21):e39633. doi: 10.1016/j.heliyon.2024.e39633 (PMC11544047; doi:10.1016/j.heliyon.2024.e39633)
Supplement: Multimedia component 1 [file mmc1.docx]

Supplementary Methods: Nanopore Sequencing and Assembly Protocol

# 1. Basecalling with Guppy

Basecalling was performed using Guppy basecaller with the following command:

guppy_basecaller -i fast5 -s fastq -c dna_r9.4.1_450bps_hac.cfg -x "cuda:0"

- Input folder (`-i`): `fast5` (containing raw fast5 files)

- Output folder (`-s`): `fastq` (for storing basecalled files)

- Configuration (`-c`): `dna_r9.4.1_450bps_hac.cfg`

- GPU mode: Enabled with `-x "cuda:0"`

# 2. Demultiplexing

Demultiplexing was performed using Guppy barcoder:

guppy_barcoder -i fastq -s barcodes -c dna_r9.4.1_450bps_hac.cfg -barcode_kits SQK-RBK110-96

# 3. Data Preparation

FASTQ files were merged for each barcode using the following command:

cat *.fastq > barcode{XX}_merged.fastq

Where `{XX}` represents the barcode numbers (1, 2, 3, …, 18, 23, 24, 25, ..., 93).

# 4. Canu Assembly

The Canu assembly pipeline was used with the following general command structure:

canu -d <output_directory> -p <prefix> \

genomeSize=<genome_size> \

maxInputCoverage=10000 \

corOutCoverage=10000 \

corMhapSensitivity=high \

corMinCoverage=0 \

redMemory=32 \

oeaMemory=32 \

batMemory=60 \

-nanopore <input.fastq>

## Genome Size Estimates

Genome sizes were estimated based on the most significant hit on species taxonomy from the EPI2ME WIMP pipeline:

- *Klebsiella pneumoniae*: 5.4m

- *Proteus mirabilis*: 4.06m

- *Escherichia coli*: 4.8m

- *Pseudomonas aeruginosa*: 6.0m

## Assembly Parameters

- `maxInputCoverage=10000`: Increases sensitivity and retains as much data as possible

- `corOutCoverage=10000`: Ensures high coverage for error correction

- `corMhapSensitivity=high`: Increases sensitivity for overlap detection

- `corMinCoverage=0`: Retains all reads for assembly

- `redMemory=32`: Memory allocation for read error detection

- `oeaMemory=32`: Memory allocation for overlap error adjustment

- `batMemory=60`: Memory allocation for best overlap graph construction (reduced from default 200 due to available system resources)

## Example Commands

canu -d mcr_colistin_osi/mk1c_colistin_020622 -p mcr_colistin_osi \

genomeSize=4.06m \

maxInputCoverage=10000 \

corOutCoverage=10000 \

corMhapSensitivity=high \

corMinCoverage=0 \

redMemory=32 \

oeaMemory=32 \

batMemory=60 \

-nanopore mcr_colistin_osi/mk1c_colistin_020622/barcode01.fastq

# Additional commands for other barcodes/organisms were executed similarly,

# adjusting the genomeSize and input FASTQ file as appropriate.

Note: The `batMemory` parameter was reduced from the default 200GB to 60GB due to system resource limitations.
